# Potential Negative Effects of Dextromethorphan as an Add-On Therapy to Methylphenidate in Children With ADHD

**DOI:** 10.3389/fpsyt.2019.00437

**Published:** 2019-06-26

**Authors:** Wei-Chen Chuang, Chin-Bin Yeh, Sheng-Chiang Wang, Pei-Yin Pan, Jia-Fwu Shyu, Yia-Ping Liu, Susan Shur-Fen Gau, Ru-Band Lu

**Affiliations:** ^1^Department of Psychiatry, Tri-Service General Hospital, National Defense Medical Center, Taipei, Taiwan; ^2^Department of Psychiatry, Tri-Service General Hospital, Songshan Branch, Taipei, Taiwan; ^3^Department of Biology & Anatomy, National Defense Medical Center, Taipei, Taiwan; ^4^Department of Physiology, National Defense Medical Center, Taipei, Taiwan; ^5^Department of Psychiatry, National Taiwan University Hospital and College of Medicine, Taipei, Taiwan; ^6^Institute of Behavioral Medicine, College of Medicine and Hospital, National Cheng Kung University, Tainan, Taiwan; ^7^Addiction Research Center, National Cheng Kung University, Tainan, Taiwan; ^8^Center for Neuropsychiatric Research, National Health Research Institutes, Miaoli, Taiwan

**Keywords:** ADHD, children, added-on therapy, methylphenidate, dextromethorphan, cytokines

## Abstract

**Objectives:** Methylphenidate (MPH) is highly effective in controlling the symptoms of attention-deficit/hyperactivity disorder (ADHD), but some children with ADHD either do not respond to, or do not tolerate, treatment. Dextromethorphan (DM) is a neuroprotective agent which has been used in the treatment of neuropsychiatric disorders. This clinical trial had examined the effect of DM on the use of MPH in the children with ADHD.

**Methods:** This randomized double-blind clinical trial had evaluated 44 male outpatients, aged between 6 and 12 years, with a diagnosis of ADHD. The study subjects were randomly assigned into one of the two groups: receiving MPH alone (15–60 mg per day) or MPH plus DM (30–60 mg per day) for 8 weeks. Assessments, comprising the Chinese version of the *Child Behavior Checklist (CBCL-C) scale and the Swanson, Nolan and Pelham Questionnaire (SNAP)-IV rating tests* conducted by parents and the serum cytokines measured by microarray and enzyme-linked immunosorband assay (ELISA), were compared between groups at baseline and at 8 weeks after the medication was started.

**Results:** There were a significant decrease at the mean scores of both CBCL-C and SNAP-IV scales after 8 weeks of treatment, but no significant differences between MPH and MPH+DM groups. Compared with the MPH-only group, the mean scores of some psychometric parameters reported on the CBCL-C and SNAP-IV scales regarding time effects as well as the attention problems on the CBCL-C scale regarding group effect were significantly higher in the DM+MPH group. Although there were no significant differences in the levels of various serum cytokines between groups, the subjects in the DM-MPH group had relatively fewer and lower levels of adverse effects. Significant interactions were found between the withdrawn/depression item reported on the CBCL-C scale and tumor necrosis factor α (ခTNF-α) (*p* = 0.027), as well as between thought problems item on the CBCL-C and TNF-α (*p* = 0.028) in subjects who had received DM+MPH treatment.

**Conclusion:** Following the trial, DM+MPH was not superior to MPH alone for the treatment of children with ADHD, yet DM may potentially have negative effects on ADHD symptoms when combined with MPH.

**Clinical Trial Registration:** Clinicaltrials.gov, trial number: NCT01787136.

## Introduction

Attention-deficit hyperactivity disorder (ADHD) affects around 5% of school-age children worldwide, and symptoms usually appear before age 12 years. Children with ADHD have a combination of persistent problems, such as inattentive, hyperactive, and impulsive behavior, which leads to academic difficulties and impairments of interpersonal functioning (1).

The exact cause of ADHD is not fully understood, although several factors may contribute to its development. Dysregulation in immunological functions is reported in the patients with ADHD. A systematic review and meta-analysis has revealed that there is a strong association between ADHD and inflammatory and autoimmune disorders (2). Several studies have anticipated the association between ADHD and inflammatory mechanisms due to strong influence of inflammation-related genes (3–6). Cytokines involved in tryptophan metabolism and dopaminergic pathways in the brain could be altered between pro-inflammatory and anti-inflammatory, influencing the pathogenesis of ADHD (6). Furthermore, the administration of interleukin-1β (IL-1β), interleukin-2 (IL-2), and interleukin-6 (IL-6) increased norepinephrine (NE) and reduced dopamine (DA) levels, which was similar to those seen in ADHD (7). Neuroinflammation could be also one of the potential mechanisms contributing to pathogenesis of ADHD (8, 9). The activation of microglia cells, the main resident immune cells of the brain, releases pro-inflammatory cytokines and other factors, such as glutamate, contributing to neuroinflammation and associates with ADHD (10).

Methylphenidate (MPH) is a DA and NE transporter blocker which enhances the dopamine transmission through eliminating DA and NE reuptake by neurons (11, 12). It has been constituted as the first-line medication in children for the short-term treatment of ADHD (12). Increasing evidence suggests that the nitric oxide (NO) produced from neuronal nitric oxide synthase (nNOS) has roles in dopamine-mediated effects of MPH and nNOS inhibitors could diminish the DA-dependent locomotor hyperactivity evoked by MPH (13). In addition, MPH-induced immune system hyperactivity has been reported, especially an increased immunoglobulin E (IgE) levels had found in children; and it could interfere the immunizations and the normal maturation of the immune system in young children (14). Although the immunological system may be involved in the mechanism of MPH, the immunologic correlation between MPH and ADHD remained unclear and the effect of MPH-mediated immunological modulation in the treatment of ADHD have not be evidently identified (12). MPH remains the U.S. Food and Drug Administration (US-FDA) approved medical treatment of choice for children with ADHD and is associated with an exceptional response rate, but around 25% of children with ADHD do not respond to MPH (15–17) and various side effects of MPH including insomnia, headaches, stomachaches, irritability, and decreased appetite have been reported (18). Therefore, the unique and individual treatment strategies that incorporate different drug treatment options should be sought.

Dextromethorphan (DM), an active ingredient used in cough suppressants. In the past decade, studies have documented that DM is a neuroprotective agent (19). It protects dopaminergic neurons through inhibition of microglia activation by reducing inflammation-mediated degeneration (8). Microglia are the central players in the neuroinflammatory process that contributed by inflammagens, such as lipopolysaccharide (LPS)-induced neurotoxicity (20, 21). The activation of microglia results in the production of proinflammatory factors, including nitric oxide (NO) (20, 22, 23), tumor necrosis factor α (TNF-α) (23, 24), interleukin-1β (IL-1β) (23, 25, 26), prostaglandin E2 (PGE2) (27), and reactive oxygen species (ROS) (28, 29), which all serve in the immune surveillance of the brain with functions to remove foreign microorganisms (30). Overproduction of pro-inflammatory factors may lead to neuronal damage in the brain (25, 31). Liu et al. had reported that DM significantly reduced the microglia-mediated degeneration of dopaminergic neurons induced by LPS and inhibited the LPS-induced production of TNF-α, NO, and superoxide free radicals (31). DM-modulated neuroinflammation might be beneficial in the treatment of neuropsychiatric disorders, including bipolar disorders (32, 33) and depression (34), schizophrenia, autistic disorders (35, 36), and seizure disorder (37).

Recent work has shown that the peripheral immune response contributes to neuroinflammatory conditions in neurological and psychiatric diseases (38). Therefore, DM with its anti-inflammation by neuroprotection may have potential to reduce ADHD symptoms by eliminating the peripheral immune response (39, 40). In addition, DM is also a well-established, uncompetitive, low-affinity *N*-methyl-*d*-aspartate (NMDA) receptor antagonist which noncompetitively blocks nicotinic (alpha-3-beta-4, alpa-4-beta-2, and alpha-7) receptors against nicotine’s antinociceptive effect (41) and also acts as an agonist at sigma-1 receptors, which are potential protein targets for antidepressant medications (41, 42). DM is a serotonin transporter and norepinephrine transporter inhibitor and has rapid-acting antidepressant effects (43, 44).

Some studies had demonstrated that the serum levels of brain-derived neurotrophic factor (BDNF), glial-derived neurotrophic factor, nerve growth factor, and neurotrophic-E were abnormal in children with ADHD (45, 46). BDNF mediates the endurance of experience-dependent changes in the brain (47) and is considered to be an important biomarker for ADHD (48). Some case reports had described that the BDNF plays a role in the reduction of problematic behaviors including ADHD symptoms among children with autism (36). In addition, the DM added-on therapy has been shown to benefit certain neuropsychiatric disorders, including bipolar disease and schizophrenia in which may be associated with its effect on modulation of BDNF (32). However, a previous study had indicated that treatment with an anti-ADHD medication did not show any direct relationship between the improvement of the severity of ADHD symptoms and the reduction of BDNF concentrations (49). Despite these protective mechanisms of DM, research exploring the detailed molecular mechanism of DM in the treatment of neuropsychiatric disorders remains unclear.

Therefore, the objective of our study was to investigate the effect of the added-on DM to MPH in the treatment of children with ADHD by measuring the internalized or externalized ADHD symptoms that most affected the children and their parents and by examining the level of serum pro-inflammatory cytokines in children.

## Methods

### Subjects

This randomized double-blind clinical trial had recruited 44 male outpatients, aged between 6 and 12 years, from Tri-Service General Hospital (Taipei, Taiwan) between January 2008 and December 2009. All the participants were diagnosed as ADHD children. This prospective study was approved by the Institutional Review Board at Tri-Service General Hospital. An informed consent was obtained from both the parents and the children prior to the commencement of the study. Registration identifier: Clinicaltrials.gov: NCT01787136.

Gender was not listed on the exclusion criteria in this study, yet all patients coincidentally happened to be male during the study period.

### Inclusion Criteria

After accessing the ADHD symptoms in patients by the Swanson, Nolan, and Pelham–IV Questionnaire (SNAP-IV), ADHD was diagnosed by a psychiatrist according to the *Diagnostic and Statistical Manual of Mental Disorders, Fourth Edition* (DSM-IV) diagnostic criteria (50, 51).

### Exclusion Criteria

The exclusion criteria were the following: 1) unwilling to participate after receiving a detailed explanation of the study; 2) could not follow the investigators’ instructions; 3) the presence of severe neurological or mental illnesses, such as epileptic disorder, or a history of stroke, schizophrenia, bipolar disorder, or mental retardation, or those who had a risk of suicide; 4) the presence of severe medical illnesses or conditions requiring surgery, or uncontrolled abnormal thyroid function, or a history of heart attack, or uncontrolled hypertension; 5) had taken antidepressants or psychotropic medications within 2 months prior to the study; 6) allergy to MPH or DM; 7) the presence of autoimmune disorders, autoimmune disorders, severe asthma attacks; 8) had severe infections currently or within 2 months prior to the beginning of the study, which may influence on the level of serum cytokines.

### Experimental Design

This randomized, placebo-controlled, double-blind pilot study was to compare the clinical efficacy of MPH alone and DM plus MPH in the treatment of children with ADHD. The severity of ADHD symptoms was rated by SNAP-IV scoring, and the level of pro-inflammatory cytokines in serum was measured by ProcartaPlex™ Multiplex Immunoassays (affymetrix eBioscience, Vienna, Austria).

### Two Study Groups

The randomization and allocation process were done. Twenty-two patients were randomly allocated to one of each group. The DM tablets were produced in the same shape, color, and weight similar as MPH. The patients were randomized to receive treatment with 15 mg to 60 mg of MPH per day plus the placebo (Group 1) or 15 mg to 60 mg of MPH plus 30 mg to 60 mg DM per day (Group 2) (52) for an 8-week double-blind clinical trial (34). For MPH+DM group, the subjects had received 30 mg DM per day for week 1 to week 2, 60 mg DM per day at week 3 to week 8, and the final dosage of DM was 60 mg daily. For MPH alone group, the subjects had received 15 mg MPH per day at week 1 to week 2, and 30 to 60 mg MPH per day at week 3 to week 8. However, the final dosage of MPH ranged from 15 to 60 mg daily, based on the child’s clinical response and the side effects, not on the forced titration.

Immediate-release MPH (Ritalin^®^; Novartis, Basel, Switzerland) was prescribed as follows: an initial dosage of 2.5 to 5 mg orally was administered twice daily, and then increased weekly in increments of 5 to 10 mg, up to a maximum of 60 mg/day in two or three divided doses (the range of titration was 0.5–1.2 mg/per kg body weight). Our dosage of MPH was adequately titrated using clinical judgment, including the response and tolerability of side effects.

During the study period, the company (TSH Biopharm, Taipei, Taiwan), who manufactured the medications, the person who administrated the medications, the manager of the study, the assistant manager, patients’ relatives, and the patients were blinded to the treatment group. The computer-generated randomization list of patients was kept in sealed and unblended until the end of this study.

## Measurements

### The Chinese Version of the Child Behavior Checklist (CBCL-C)

The Chinese CBCL is a parent-reported questionnaire concerning their children aged 4 to 18 years. The CBCL/4-18 (51) measures 20 areas of competence and 118 behavioral/emotional items, using a three-point Likert scale. The form can also be administered orally by an interviewer who then records the parent’s or caregiver’s answers. To avoid improper scoring, there are several items for which the respondent is asked to elaborate about an endorsed behavior. Some syndromes were combined to provide scores for broad-band internalizing problems (the sum of withdrawn, somatic complaints, and anxious/depressed) and externalizing problems (the sum of delinquent behavior and aggressive behavior).

The T score for each behavioral/emotional problem of the Chinese CBCL was generated based on well-established normative data in Taiwan (51, 53). A T score of 50 in each subscale indicates average functioning in reference to other children of the same age and gender. A T score greater than 67 was used as the cutoff point to define the presence of each extreme behavioral/emotional problem.

### The SNAP-IV Scale

The SNAP-IV scale (54) was as used for the assessment of the severity of ADHD. The instrument has four subscales (inattention, 9 items; hyperactivity/impulsivity, 9 items; and oppositional, 8 items). SNAP-IV items are rated on a four-point scale, from 0 to 3. This measure has been frequently used in ADHD investigations, including those designed to assess clinical intervention; in one study, use of the SNAP-IV ratings increased the accuracy of ratings by 30% (55).

In this study, the SNAP-IV was completed by the subjects’ parents or guardians. The treatment response for both groups was judged as good or poor, depending on the improvement in the SNAP-IV score of the individual. Good responders were defined as those who had a greater than 50% improvement in their SNAP scores, whereas poor responders were defined as those who had less than 50% improvement in their SNAP-IV scores.

### Pro-Inflammatory Cytokines

Serum was isolated from blood collected before and after 8 weeks of treatment from patients. The serum was simultaneously analyzed for the following cytokines: granulocyte-macrophage colony-stimulating factor (GM-CSF), interferon gamma (IFN-γ), interleukin-1 beta (IL-1 beta), IL-2, IL-4, IL-5, IL-6, IL-12, IL-13, IL-18, and TNF-α by the protein microarray system, ProcartaPlex^™^ Multiplex Immunoassays (affymetrix eBioscience, Vienna, Austria). Briefly, following the manufacturer’s guidelines, once antigen reagents and serum samples were prepared, all were incubated with antibody-coated beads for 2 h in individual wells of a 96-well plate. Then, detection antibodies were added in each well and incubated for 30 min. After this, streptavidin R-phycoerythrin solution was added and incubated in each well for 30 min. After adding the reading buffer, the data were read by a Luminex^®^ 100 system analyzer (ThermoFisher Scientific, Waltham, MA, USA). All incubates were stored in darkness and kept at room temperature.

### Statistical Analysis

The data were accumulated, analyzed, and presented as mean and standard deviation (mean ± SD). The comparisons between different treatments were analyzed by independent *t*-tests. Due to the repeated measurement the scores of the CBCL-C and SNAP-IV tests and cytokine measurements were subjected to change, a linear model was used to investigate the effect of groups (Group Effect), test times (Time Effect), and their interaction (Group × Time Effect). When the main effects or interactions were found to be significant, Bonferroni corrections were used for controlling type I errors during *post hoc* multiple comparisons. For cytokine measurements, cytokine detection or lack of detection and significant differences between groups were adjusted in that model. The repeated-measures analysis of variance (ANOVA) was used to detect the interaction between withdrawn/depression (or thought problems) of CBCL-C and TNF-α. Statistical analyses were performed with IBM SPSS statistical software version 22 for Windows (IBM Corp, Armonk, NY, USA), and a two-tailed *p* < 0.05 was considered statistical significance.

## Results

### Baseline Levels on CBCL-C and SNAP-IV, and Cytokines

**Figure 1:**
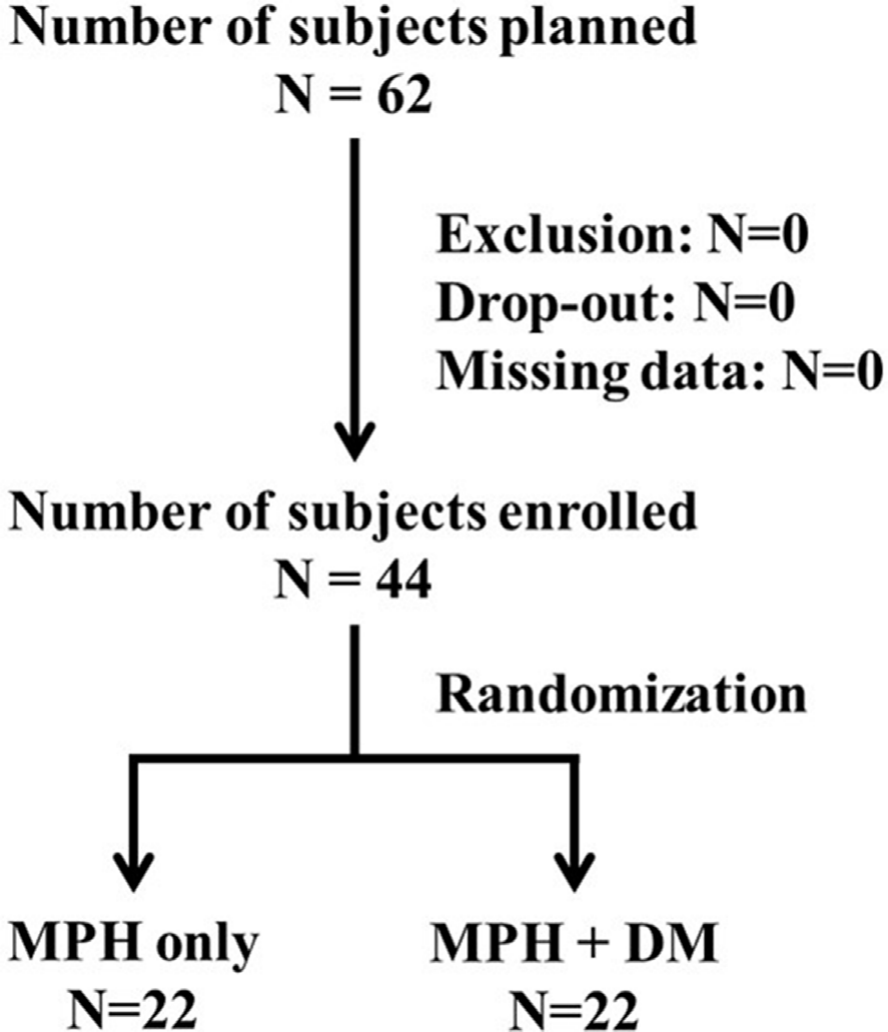
Flow Diagram of Participants.

We had initially planned to recruit 62 subjects based on the power calculation (G power: set alpha = .05, power = 0.8, effective size = 0.3, total *N* = 62); however, only 44 patients were enrolled in the study due to the recruitment difficulty of children in Taiwan (**Figure 1**). All subjects were male. The median ages of the subjects were 9.16 (±1.71) and 9.27 (±1.73) years in MPH only and DM+MPH groups, respectively (*P* = 0.833) (**Table 1**).

**Table 1:** The age, mean values of the Chinese version of the Child Behavior Checklist (CBCL-C) and Swanson, Nolan and Pelham Questionnaire (SNAP)-IV scores, and serum cytokines levels of the patients in both groups at baseline.

	MPH only (*n* = 22)	DM-MPH (*n* = 22)	*p*-value
Age (yr)	9.16 ± 1.71	9.27 ± 1.73	0.833
CBCL-C scores			
Anxiety/depression	6.09 ± 5.26	6.73 ± 4.51	0.669
Withdrawn/depression	3.18 ± 2.06	2.68 ± 2.46	0.469
Somatic complaints	2.68 ± 3	2.09 ± 2.64	0.491
Social problems	5.32 ± 2.92	6.77 ± 4.58	0.217
Thought problems	4.18 ± 2.86	4 ± 2.67	0.828
Attention problems	10.73 ± 3.31	11.91 ± 3.45	0.253
Delinquent behavior	5.27 ± 4.1	4.95 ± 2.68	0.762
Aggressive behavior	10.95 ± 6.28	12.82 ± 7.69	0.384
Other problems	6.77 ± 3.34	6.91 ± 2.83	0.884
SNAP-IV scores			
Inattention subscore	15.09 ± 5.24	15.91 ± 4.95	0.597
Hyperactivity subscore	12.41 ± 5.8	13.18 ± 6.77	0.686
Oppositional defiant subscore	10.36 ± 5.7	11.82 ± 4.94	0.371
Cytokines			
IL-12p70	21.27 ± 24.87	26.97 ± 14.23	0.356
IFN-γ	127.49 ± 269.75	131.76 ± 242.22	0.956
IL-17A	703.88 ± 1341.18	666.96 ± 789.98	0.912
IL-2	242.77 ± 406.56	662.8 ± 1245.37	0.140
IL-10	27.1 ± 50.32	25.69 ± 26.33	0.908
IL-9	331.47 ± 535.72	1,805.89 ± 6164.57	0.270
IL-22	681.09 ± 447.59	635.11 ± 326.23	0.699
IL-6	5.47 ± 10.55	7.46 ± 12.59	0.572
IL-13	72.56 ± 85.44	111.92 ± 129.07	0.240
IL-4	101.78 ± 146.95	109.38 ± 64.57	0.826
IL-5	30.42 ± 33.83	63.15 ± 107.8	0.181
IL-1α	64.09 ± 79.86	74.77 ± 70.99	0.642
TNF-α	28.13 ± 61.52	37.19 ± 59.07	0.621

The MPH only and DM+MPH groups were similar on their mean CBCL-C scores, SNAP scores, and level of serum different cytokines before the treatment (all *p* > 0.05) (**Table 1**).

### CBCL-C and SNAP-IV Scores Pre- and Post-Treatment

As shown in **Table 2**, the mean CBCL-C and SNAP-IV scores were significantly decreased in both groups at week 8 compared with the baseline yet not significantly different between the groups. For the *post hoc* multiple comparisons, the mean values for attention problems (10.73 vs. 7, *p* = 0.002), aggressive behavior (10.95 vs. 6.18, *p* = 0.014), and other problems (6.77 vs. 4.41, *p* = 0.009) reported on the CBCL-C scale and all the core symptoms (inattention: 15.09 vs. 9.68, *p* = 0.002; hyperactivity: 12.41 vs. 6.55, *p* = 0.004; oppositional defiant: 10.36 vs. 6.77, *p* = 0.002) on SNAP-IV scale were significantly reduced after an 8-week treatment in the MPH-only group. In the DM+MPH group, the mean values for social problems (4.59 vs. 6.77, *p* = 0.049), attention problems (8.86 vs. 11.91, *p* = 0.017), delinquent behavior (2.73 vs. 4.95, *p* = 0.025), aggressive behavior (8.27 vs. 12.82, *p* = 0.019), and other problems reported on the CBCL-C scale and the scores for inattention (15.91 vs. 10.14, *p* < 0.001) and hyperactivity (13.18 vs. 7.82, *p* < 0.001) on SNAP-IV scale were significantly reduced after an 8-week treatment (**Table 2**).

**Table 2:** Comparison of the changes in CBCL-C and SNAP-IV scores between treatment groups at pre- and post-treatment.

	MPH-only (*p* = 22)			DM-MPH (*p* = 22)					
	Pre-treatment	Post-treatment	*p*-value between pre- and post-treatment	Pre-treatment	Post-treatment	*p*-value between pre- and post-treatment	*p*-value for time effect	*p*-value for group effect	*p*-value for interaction
CBCL-C									
Anxiety/depression	6.09 ± 5.26	3.68 ± 3.81	0.450	6.73 ± 4.51	4.45 ± 4.01	0.556	0.015^†^	0.458	0.943
Withdrawn/depression	3.18 ± 2.06	2.18 ± 2.58	0.973	2.68 ± 2.46	2.41 ± 2.28	1.000	0.208	0.786	0.470
Body complaint	2.68 ± 3	2.18 ± 3.1	1.000	2.09 ± 2.64	1.36 ± 1.79	1.000	0.286	0.221	0.843
Social problems	5.32 ± 2.92	3.23 ± 2.96	0.358	6.77 ± 4.58	4.59 ± 3.81*	0.0496	0.007^†^	0.072	0.953
Thought problems	4.18 ± 2.86	2.64 ± 2.11	0.336	4 ± 2.67	3.41 ± 2.87	1.000	0.062	0.602	0.400
Attention problems	10.73 ± 3.31	7 ± 3.09*	0.002	11.91 ± 3.45	8.86 ± 3.24*	0.017	<0.001^†^	0.032^†^	0.627
Delinquent behavior	5.27 ± 4.1	3.55 ± 3.53	0.487	4.95 ± 2.68	2.73 ± 2.39*	0.025	0.005^†^	0.414	0.719
Aggressive behavior	10.95 ± 6.28	6.18 ± 5.84*	0.014	12.82 ± 7.69	8.27 ± 5.11*	0.019	0.001^†^	0.145	0.933
Other problems	6.77 ± 3.34	4.41 ± 2.67*	0.009	6.91 ± 2.83	5.64 ± 2.82	0.915	0.005^†^	0.277	0.384
SNAP									
Inattention subscore	15.09 ± 5.24	9.68 ± 5.41*	0.003	15.91 ± 4.95	10.14 ± 4.37*	0.002	<0.001^†^	0.553	0.865
Hyperactivity subscore	12.41 ± 5.8	6.55 ± 4.14*	0.004	13.18 ± 6.77	7.82 ± 5.11*	0.011	<0.001^†^	0.389	0.833
Oppositional defiant subscore	10.36 ± 5.7	6.77 ± 5.06*	0.022	11.82 ± 4.94	8.91 ± 4.58	0.368	0.004^†^	0.102	0.754

*p < 0.05; significantly different between pre- and post-treatment in post hoc multiple comparisons.

^†^p < 0.05; significant difference in Time effect, Group effect.

The time effect was defined as the differences before and after treatment between different groups. A linear regression analysis had showed that there were significant differences between groups regarding Time Effect in anxiety/depression, social problems, attention problems, delinquent behavior, aggressive behavior, and other problems reported on the CBCL-C scale and all the core symptoms on SNAP-IV scale (*p* ≤ 0.015) (**Table 2**), yet the mean scores of these items were significantly higher in the DM+MPH group compared to the MPH only one. The Group Effect reflected the differences between the subjects who received treatment with MPH only and those with DM+MPH. The significant difference between groups regarding Group Effect was found only in attention problems on the CBCL-C scale (*p* = 0.032) not on other items, but the mean value of this item was significantly higher in the DM+MPH group compared to the MPH only one. In the relation to the interactions indicating the changes of scores on all CBCL-C and SNAP-IV items pre- and post-treatment between groups, there were no significant differences between groups (**Table 2**).

### Cytokines Levels Pre- and Post-Treatment

In our study, we found that the detection of certain cytokines was challenging and vary between groups, so that cytokines including IFN-γ, IL-2, IL-10, IL-9, IL-13, and TNF-α were adjusted for the detectability. According to **Table 3**, there were no significant differences in levels of various serum cytokines between groups analyzed by *post hoc* multiple comparisons and also at time effect, group effect, and the interaction between the two effects examined by linear regression analyses (all *p* > 0.05).

**Table 3:** Comparison the changes in serum concentrations of various cytokines between treatment groups at pre- and post-treatment.

	MPH-only (*n* = 22)			DM-MPH (*n* = 22)					
	Pre-treatment	Post-treatment	*p*-value between pre- and post-treatment	Pre-treatment	Post-treatment	*p*-value between pre- and post-treatment	*p*-value for time effect	*p*-value for group effect	*p*-value for interaction
Cytokine									
IL-12p70	21.27 ± 24.87	19.55 ± 15.74	1.000	26.97 ± 14.23	20.66 ± 14.93	1.000	0.297	0.377	0.551
IFN-α^†^	127.49 ± 269.75	83.09 ± 149.55	1.000	131.76 ± 242.22	285.87 ± 858.41	1.000	0.587	0.306	0.327
IL-17A	703.88 ± 1341.18	526.07 ± 916.17	1.000	666.96 ± 789.98	493.75 ± 608.91	1.000	0.39	0.865	0.991
IL-2^†^	242.77 ± 406.56	274.51 ± 450.2	1.000	662.8 ± 1,245.37	947.69 ± 2,903.59	1.000	0.646	0.115	0.713
IL-10^†^	27.1 ± 50.32	27.68 ± 53.78	1.000	25.69 ± 26.33	25.4 ± 43.77	1.000	0.988	0.848	0.964
IL-9^†^	331.47 ± 535.72	256.63 ± 531.5	1.000	1,805.89 ± 6,164.57	1,796.7 ± 6,152.82	1.000	0.964	0.11	0.972
IL-22	681.09 ± 447.59	615.56 ± 333.47	1.000	635.11 ± 326.23	621.21 ± 421.59	1.000	0.631	0.807	0.755
IL-6	5.47 ± 10.55	5.09 ± 8.64	1.000	7.46 ± 12.59	8.8 ± 16.6	1.000	0.858	0.286	0.748
IL-13^†^	72.56 ± 85.44	74.3 ± 86.5	1.000	111.92 ± 129.07	87.05 ± 73.92	1.000	0.574	0.207	0.518
IL-4	101.78 ± 146.95	87.94 ± 80.05	1.000	109.38 ± 64.57	154.57 ± 325.29	1.000	0.693	0.351	0.458
IL-5	30.42 ± 33.83	30.07 ± 34.06	1.000	63.15 ± 107.8	50.66 ± 100.51	1.000	0.699	0.11	0.714
IL-1b	64.09 ± 79.86	71.41 ± 84.28	1.000	74.77 ± 70.99	70.85 ± 78.09	1.000	0.919	0.763	0.738
TNF-α^†^	28.13 ± 61.52	30.18 ± 59.72	1.000	37.19 ± 59.07	25.25 ± 42.81	1.000	0.681	0.864	0.561

^†^Adjusted for detectability, since the ability to detect these cytokines was significantly different between groups.

### Adverse Effects Pre- and Post-Treatment

As is shown in **Table 4**, all patients had mild level of adverse effects. There were no significant differences in the occurrence and severity of adverse effects included somnolence, gastric discomfort, headache, insomnia, and poor appetite between patients with MPH only or MPH+DM treatments (*p* > 0.05). However, there was a relatively lower level of adverse effects and no report in children with moderate level of headache or insomnia and fewer children with moderate level of gastric discomfort or poor appetite in the DM+MPH group compared to the MPH only group.

**Table 4:** Comparison of the adverse events between the two groups.

	MPH only (*n* = 22)	DM-MPH (*n* = 22)	*p*-value
Somnolence			1.000
Mild	21 (95.5%)	21 (95.5%)	
Moderate	1 (4.5%)	1 (4.5%)	
Gastric discomfort			1.000
Mild	19 (86.4%)	20 (90.9%)	
Moderate	3 (13.6%)	2 (9.1%)	
Headache			0.233
Mild	19 (86.4%)	22 (100%)	
Moderate	3 (13.6%)	0 (0%)	
Insomnia			0.108
Mild	18 (81.8%)	22 (100%)	
Moderate	4 (18.2%)	0 (0%)	
Poor appetite			0.698
Mild	17 (77.3%)	19 (86.4%)	
Moderate	5 (22.7%)	3 (13.6%)	

### Interaction between Withdrawn/Depression or Thought Problems and TNF-α in the DM+MPH Group

We had found that there were significant interactions between the withdrawn/depression item reported on the CBCL-C scale and TNF-α (*p* = 0.027) and between the thought problems item on the CBCL-C scale and TNF-α (*p* = 0.028) in subjects who had received DM-MPH treatment (data not shown).

## Discussion

Our study had revealed that there were no significant differences at age and baseline profile of 44 male children with ADHD in the DM+MPH and MPH only groups. After 8 weeks of treatment, the significant changes in some psychometric parameters of ADHD were observed in both groups. Compared to the MPH only group, the mean scores of certain symptom items reported on the CBCL-C and SNAP-IV scales regarding time effects and the attention problems item on the CBCL-C scale regarding group effect were higher in the DM+MPH group. Although there were no significant differences in levels of various serum cytokines between groups, the subjects in the DM+MPH group have relatively fewer and lower level of adverse effects. Significant interactions were found between the withdrawn/depression items on the CBCL-C and TNF-α, as well as thought problems on the CBCL-C and TNF-α in subjects who had received DM+MPH treatment.

The Chinese versions of CBCL and the SNAP-IV have been shown to have good reliability and validity (53, 56). Yang et al. had compared the internal consistency and 1-month test–retest reliability of the CBCL-C and the SNAP-IV scales among 852 Taiwanese junior high school students at 12 to 16 years of age and found that both tests were effective for evaluating the students’ mental health (56). However, the CBCL-C scale has a cross-cultural generalizability in many societies, including Taiwan (57). Chang et al. had indicated that CBCL-C scale equipped with good sensitivity and specificity and was recommended for detecting comorbid conditions of ADHD, which often caused problematic symptoms for the patients and their family (58). Wang and colleagues also reported the strengths of using multiple tests with different observers to study patients with ADHD and showed that multiple tests administered in a clinical setting may help identify behavioral changes among patients with ADHD, thereby providing a clearer view of an ADHD patient’s true status (59). The SNAP-IV also has high discriminant validity by clearly distinguishing children with ADHD (60). These two approaches were employed to identify and measure the psychometric properties of ADHD in this study.

In this study, the mean values for attention problems, aggressive behavior, and other problems reported on the CBCL-C scale and all the core symptom items on SNAP-IV scale were significantly reduced after an 8-week treatment in the MPH only group. In the DM+MPH group, the mean values for social problems, attention problems, delinquent behavior, aggressive behavior, and other problems reported on the CBCL-C scale and the scores for inattention and hyperactivity on SNAP-IV scale were significantly reduced after an 8-week treatment. This result had suggested that DM might benefit cognitive functions other than the behavioral or emotional manifestations reported among children with ADHD. Previous studies had found that immature working memory may be associated with the inattention symptoms of children with ADHD (61, 62). The attention problems might be modified by the mechanisms of neuroprotection (39, 63) or cognitive enhancement (64). Therefore, the results of our study had indicated that it may be critical to observe the cognitive performance variations affected by possible cognitive modifiers in the treatment of children with ADHD.

Mitchell and Goldstein had conducted a systematic review in the inflammation and neuropsychiatric disorders in children and adolescents which analyzed 67 studies including a total of 3,952 youths and indicated that there was a proinflammatory state for autism spectrum disorders (65). A systematic review on data from 14 manuscripts had showed variable results regarding the association between inflammation and ADHD (66), and the other study had stated there is no clear evidence in the inflammation involved in ADHD pathophysiology (67). Despite a number of studies that have shown that DM has a potent immunomodulatory capacity, suggesting it is a useful adjutant drug for psychiatric disorders, such as autism and schizophrenia (36), we have found a negative effect of DM on the use of MPH in the treatment of ADHD in children.

In our study, we found that the subjects in the DM+MPH group have relatively fewer and lower level of adverse effects. DM is an uncompetitive, low-affinity NMDA receptor antagonist and coupled with the high-affinity agonist activity at sigma-1 receptors, clinical improvements in neurobehavioral disorders might be attributed to neuroprotection against glutamate excitotoxity (68) and the immunomodulatory effects on the inhibition of serotonin and norepinephrine transporters (42). These effects, however, appear to be marginal and failed to support the efficacy of DM in treating ADHD symptoms in children.

The potential drug–drug interactions between MPH and DM have yet to be explored. A study using human liver microsomes (69) had reported that MPH did not inhibit DM’s actions, and MPH was unaffected by DM. We found that there were no significant differences in levels of various serum cytokines between groups. Proinflammatory markers were elevated in several other neuropsychiatric disorders, including major depressive disorder, post-traumatic stress disorder, obsessive-compulsive disorder, Tourette syndrome, ADHD, and schizophrenia, yet the data were inconsistent, and the evidence was preliminary (65). The ADHD patients had increased serum levels of IL-6, IL-10, antibasal ganglia antibodies, and antibodies against the dopamine transporter, supporting the role of the immune system in the disorder; however, no significant results were found with regard to the evaluated serum IL-6 and TNF-α in a different study (67). Therefore, the mixed results were reported in relation to the evaluation of serum levels of inflammatory markers in ADHD patients, likely due to small sample sizes and a high heterogeneity between biomarkers (67).

TNF-α is a potent enhancer of inflammatory reactions and induces the expression of downstream inflammatory factors (70). It also triggers the upregulation of IL-6 and mediates multiple biological effects (71). We found that there were significant interactions between the withdrawn/depression items on the CBCL-C and TNF-α, as well as between the thought problems item on the CBCL-C and TNF-α in subjects who had received DM-MPH treatment. Several studies had indicated that DM inhibits several inflammatory processes by suppressing the induction of pro-inflammatory cytokines (31, 72). The present study had established a direct causal association between proinflammatory cytokines and withdrawn/depression, preclinical and clinical evidence show that the peripheral administration of pro-inflammatory cytokines, such as TNF-α, can prompt depression-like behavior (73).

Our study had several limitations. It was a small prospective study conducted at a single treatment center. The appropriate sample size should be 62 to reach G power (set alpha = .05, power = 0.8, effective size = 0.3) and 30 subjects for each arm for accessing the significant differences between proinflammatory cytokines two groups. However, only 44 children were included, the statistical power in this study may be not adequate to detect a meaningful difference between groups after treatment at this stage. The tic disorder, autism, or pain syndromes also have not been directly addressed in our study. In addition, it is difficult to generalize our findings; due to the fact that the subjects were all male children in this study made it difficult to generalize our findings to girls.

## Conclusion

The antitussive dextromethorphan has the immunomodulatory capability, suggesting that it may be effective for a variety of psychiatric and pain syndromes, particularly neurobehavioral disorders of childhood such as autism and ADHD. Although our results had suggested that DM may potentially have negative effects when combined with MPH on the treatment of children with ADHD, it had improved symptoms including withdrawn/depression and thought problems of children. Further studies on the long-term effects and efficacy of the DM added on therapy with MPH incorporating serum cytokines changes should be investigated in larger groups of children with ADHD.

## Ethics Statement

This double-blind, randomized controlled clinical trial was approved by the Institutional Review Board of Tri- Service General Hospital. Both the children and the parents/legal guardians of children participants have provided written informed consent in the study.

## Author Contributions

CY: Guarantor of integrity of the entire study, study concepts, study design, definition of intellectual content. WC: Clinical studies. SW: Definition of intellectual content. PP: Experimental studies, data acquisition. JS: Experimental studies, data acquisition. YL: Experimental studies, data acquisition, data analysis. SG: Manuscript editing, manuscript review, statistical analysis. RL: Manuscript editing, manuscript review.

## Funding

We thank the research funding from TSGH-C106-103, TSGH-C107-105 and Teh-Tzer Study Group for Human Medical Research Foundation.

## Conflict of Interest Statement

The authors declare that the research was conducted in the absence of any commercial or financial relationships that could be construed as a potential conflict of interest.
